# Fast ultraviolet-C photonics: generating and sensing laser pulses on femtosecond timescales

**DOI:** 10.1038/s41377-025-02042-2

**Published:** 2025-11-19

**Authors:** Benjamin T. Dewes, Tim Klee, Nathan D. Cottam, Joseph J. Broughton, Mustaqeem Shiffa, Tin S. Cheng, Sergei V. Novikov, Oleg Makarovsky, John W. G. Tisch, Amalia Patané

**Affiliations:** 1 School of Physics & Astronomy, University of Nottingham, Nottingham, UK; 2 Blackett Laboratory, Imperial College London, London, UK

**Keywords:** Ultrafast lasers, Optical properties and devices, Optoelectronic devices and components

## Abstract

Photonic devices operating in the ultraviolet UV-C range (100–280 nm) have diverse applications from super-resolution microscopy to optical communications, and their advances promise to unlock new opportunities across science and technology. However, generating and detecting ultrafast light signals in this spectral range remains a major challenge. Here, we report an integrated UV-C source-sensor platform that combines phase-matched second-order processes in nonlinear optical crystals for the efficient generation of femtosecond UV-C laser pulses with a new class of room temperature photodetectors based on two-dimensional (2D) semiconductors. Unexpectedly, these 2D sensors exhibit a linear to super-linear photocurrent response to pulse energy, a highly desirable property, laying the foundation for UV-C-based photonics operating on femtosecond timescales over a wide range of pulse energies and repetition rates. As proof of concept, we demonstrate a free-space communication system.

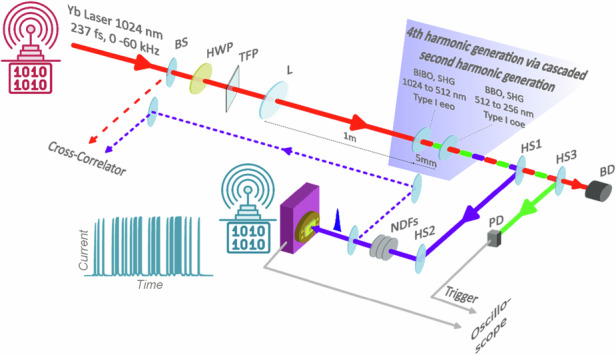

## Abstract (web summary)

A UV-C source-sensor communication system combines phase-matched second-order processes in nonlinear optical crystals for generation of femtosecond UV-C laser pulses with fast-sensors based on two-dimensional semiconductors.

## Introduction

The ability to generate and detect light in the UV-C range (wavelengths 100–280 nm, photon energies 12.4–4.4 eV) is crucial for a wide range of scientific and technological applications. The short wavelength of UV-C light enables super-resolution microscopy, allowing nanoscale imaging^1^. Its strong absorption by materials makes it indispensable for material processing applications, including surface activation, cleaning, and photolithography^2^. UV-C light is also widely used for sterilization, disrupting microbial DNA and RNA and preventing replication, making it an essential tool for water, air and surface disinfection^3,4^. In the biomedical field, it enables high-contrast imaging and precise targeting of biological tissues due to its strong interaction with organic molecules^5^. Short-pulse UV-C light is also crucial for studying ultrafast molecular dynamics, ionization processes and nonlinear optical effects — often inaccessible at longer wavelengths — enabling precise probing of fast-evolving processes and complex interactions^6,7^. Additionally, UV-C light is widely used in spectroscopy and environmental monitoring, where it facilitates the detection and quantification of trace substances through unique fluorescence and absorption signatures^8^. Beyond these applications, UV-C’s strong atmospheric scattering properties open new possibilities in non-line-of-sight communication systems, e.g., enabling data transmission in obstructed environments^9^.

Despite its vast potential, the widespread adoption of UV-C technology remains limited by lack of suitable photonic components. While lasers have advanced significantly, UV-C lasers are still scarce. Commercial excimer lasers^10^ allow for high average powers and a variety of UV-C wavelengths, but suffer from bulky footprints and high power consumption^11^. Wide bandgap diode lasers (e.g., AlGaN), while compact and efficient, are not yet commercially available and only possess a low optical output power^12,13^. UV-C light-emitting diodes (LEDs) have emerged as a promising, energy-efficient alternative^14^. However, their low external quantum efficiencies and limited optical output power currently restrict their use in applications requiring high intensities, while their inherent incoherence and challenges in beam collimation and divergence limit their suitability for applications that demand precise beam qualities. A more versatile approach involves nonlinear frequency conversion^15,16^, where solid-state lasers emitting in the near-infrared (NIR) or visible (VIS) range drive nonlinear processes in crystals to generate UV-C light. These systems can, in principle, produce ultrashort pulses with durations as low as 10 fs^17^ and low-divergence UV-C beams, making them well-suited for many applications. However, only a limited number of nonlinear crystals are suitable for UV-C operation, driving ongoing research into new candidates^18,19^. Further efforts are needed to develop compact and efficient sources based on this approach, particularly to leverage emerging high-repetition rate, low-pulse-energy lasers^20^.

In parallel, advances in UV-C detection are shaping the field^21^. Wide-band gap semiconductors and the growing class of two-dimensional semiconductors (2DSEM) with absorption in the UV-C^22^ are opening new possibilities. The latter include the wide bandgap hexagonal boron nitride (hBN)^23–25^, 2D metal chalcogenide (MC) compounds^26,27^, and hybrid heterostructures that combine graphene with a UV-C absorbing 2DSEM^28,29^. Compared to traditional semiconductors or existing technologies for UV-C sensing (e.g., photomultiplier tubes and Si photodiodes), 2DSEM-based detectors have the advantage of being compatible with different sensing platforms, including flexible and lattice mismatched substrates while operating at low applied voltages. However, most 2DSEM materials are still obtained via top-down fabrication techniques, such as mechanical exfoliation of bulk crystals. While wafer-scale growth is advancing, significant challenges remain in developing scalable UV-C sensors with high signal-to-noise ratios, broad frequency bandwidths, and precise spectral selectivity. This has led to increasing research into advanced manufacturing techniques^28,30^.

**Fig. 1:**
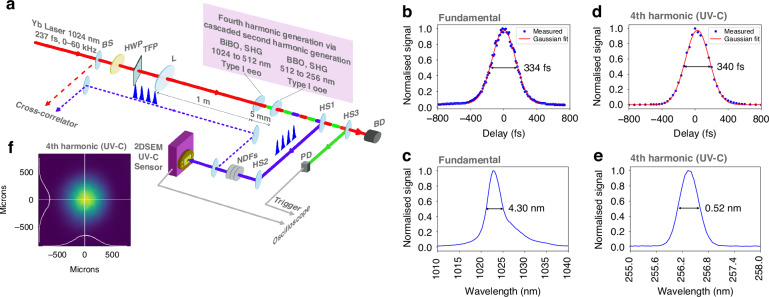
Generation and detection of fs UV-C laser pulses. **a** Experimental setup. Fourth harmonic (FH) pulses at 256 nm are generated by cascaded second harmonic (SH) generation of 236 fs pulses from a 1024 nm Yb laser. Beam splitter (BS) directs a small fraction of the fundamental (1024 nm) to be used in cross-correlation. A half-wave plate (HWP) and thin-film polariser (TFP) allow for control over the fundamental pulse energy. Lens (L) focuses the fundamental pulses onto a BiBO crystal to generate the SH at 512 nm. This in turn generates the FH at 256 nm in a BBO crystal. Harmonic separators (HS1, HS2) separate the FH from the SH and fundamental. Neutral density filters (NDF) allow for control of the FH pulse energy incident on the 2DSEM sensor. The SH at 512 nm is sent to Si photodiode (PD) via a HS3 to provide electrical triggers for a sampling oscilloscope used to measure the sensor signal. The remaining fundamental is dumped in a beam dump (BD). **b** Autocorrelation trace of fundamental with 334 fs FWHM, corresponding to 236 fs FWHM pulse duration. **c** Fundamental spectrum centred at 1024 with 4.3 nm FWHM. **d** Cross-correlation trace of FH with 340 fs FWHM, corresponding to 243 fs pulse duration. **e** FH spectrum centred at 256 with 0.52 nm FWHM. **f** FH focal spot measurement in plane of the sensor showing a near Gaussian intensity profile with 1/*e*^2^-intensity diameter of 890 μm

Here, we introduce a new platform for the generation and detection of UV-C laser pulses (Fig. 1). Our system integrates ultrafast UV-C laser source with scalable, high quality 2DSEM-based UV-C sensors. The source exploits phase-matched second-order nonlinear processes via cascaded second harmonic (SH) generation in nonlinear crystals to generate the fourth harmonic (FH) of a femtosecond laser, producing UV-C pulses with energies up to ≈ 2 μJ. We detect these pulses at room temperature by photodetectors based on the 2DSEM gallium selenide (GaSe) and its wide bandgap oxide layer (e.g., Ga_2_O_3_) with enhanced UV-C absorption. We observe a linear and super-linear photocurrent response to laser energy. This differs from that reported in sensing experiments with continuous wave (cw) lasers and represents a highly desirable property for UV-C photonics. Furthermore, all materials are compatible with scalable manufacturing processes, paving the way for a new generation of UV-C photonic applications. As proof of concept, we demonstrate a free-space communication system: a message is encoded by the fs laser source-transmitter and decoded by the 2D sensor-receiver.

## Results

### Generation and detection of fs UV-C laser pulses

Figure 1 a shows the integration of the UV-C femtosecond laser source with the 2DSEM sensor. Femtosecond UV-C pulses are generated by cascaded second harmonic generation (SHG) of a 1024 nm laser (Carbide CB5, Light Conversion) in nonlinear crystals to produce the FH at 256 nm. Bismuth triborate (BiBO) and beta-barium borate (BBO) were chosen for their suitability for SHG in the VIS and UV ranges^31,32^. The laser (fundamental) has a pulse duration of 236 fs and spectral width of 4.3 nm (Fig. 1b, c, respectively) and a variable pulse repetition rate *f*_rep_ adjustable from 0 to 60 kHz, with a maximum pulse energy of 100 μJ. The laser beam passes through a half-wave plate and thin-film polariser, which allow power control by rotating the waveplate. The vertically polarised beam is then focused by a 1 m focal length lens into a 1 mm-thick anti-reflection coated BiBO crystal. This is cut at an angle of 166.6^∘^ for type 1 eeo phase-matched SHG to produce light at 512 nm. A 0.3 mm-thick BBO crystal, placed 5 mm downstream of the BiBO crystal and within the Rayleigh range, is used to double the 512 nm light to 256 nm in the second SHG step. The BBO is cut at an angle of 50^∘^ for type 1 ooe phase-matched SHG.

Harmonic separators are used to reduce residual 1024 and 512 nm light by a factor ≈ 4.4 × 10^3^ relative to the 256 nm light. With this setup the maximum conversion efficiency of FH (from 1024 to 256 nm) of 20% is achieved for a fundamental pulse energy of 11.8 μJ at *f*_rep_ = 60 kHz, corresponding to a UV-C pulse energy of 2.38 μJ after the harmonic separators. To the best of our knowledge, this is the highest reported efficiency for these laser parameters. Furthermore, this is achieved using a relatively compact, portable laser system.

The duration of the UV-C pulse is measured by cross-correlation with the fundamental pulse^33^. A beam-splitter placed directly after the laser splits off part of the fundamental beam for this measurement. Figure 1d, e show the cross-correlation trace and the UV-C pulse spectrum, respectively. The spectral full width at half maximum (FWHM) is 0.52 nm and the cross-correlation FWHM is 340 fs. This corresponds to a UV-C pulse duration of 243 fs, assuming Gaussian pulse shapes. The UV-C beam incident on the 2DSEM sensor (located 1 m from the BBO crystal) has a near Gaussian intensity distribution (Fig. 1f) with a diameter of 890 μm (at the *e*^−2^ intensity points), comparable to the 1 mm^2^ sensor active area *A*. A set of UV-C-compatible neutral density filters is used to control the UV-C pulse energy delivered to the sensor. The 512 nm beam from the second harmonic separator is sent to a Si photodiode to provide an electronic trigger for the sampling oscilloscope used to measure the sensor output. For further details of the UV-C source, including choice of BiBO and BBO crystals, see Methods and Fig. S1 in Supplementary Note 1.

### Photoactive UV-C two-dimensional semiconductors

**Fig. 2:**
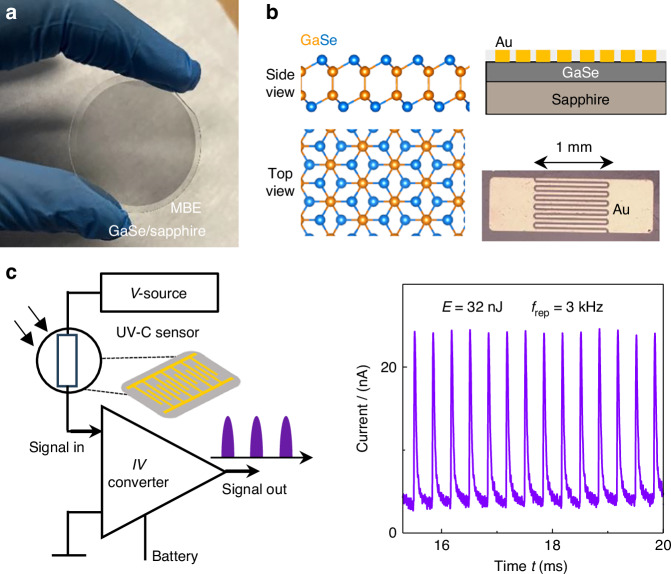
Semiconductors for UV-C sensing. **a** Image of GaSe grown by MBE on a 2 inch sapphire wafer. **b** Left: Side view and top view of the GaSe crystal lattice. Right: Schematic and optical image of the GaSe/sapphire sensor with Au electrodes (finger spacing of 50 μm and sensing area *A* = 1 mm^2^). **c** Left: Diagram of the measurement scheme used for detection of fs UV-C pulses by the sensor. Right: Electrical pulses generated by the sensor in response to a train of fs UV-C (256 nm) laser pulses with energy *E* = 32 nJ and repetition rate *f*_rep_ = 3 kHz

**Fig. 3:**
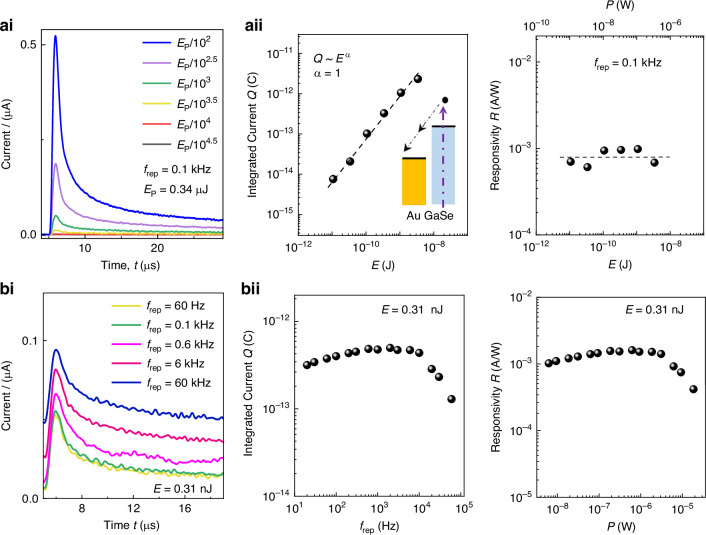
Detection of fs UV-C laser pulses by GaSe/sapphire. **ai** Response of GaSe (layer thickness of 50 nm) to a single pulse within a train of pulses at different energies *E* and repetition rate *f*_rep_ = 0.1 kHz in vacuum (*V* = 10 V). **aii** Left: Integrated current *Q* = ∫*I*(*t*) *dt* versus *E*. The dashed line describes a linear dependence of *Q* on *E*. Inset: Schematic of the photo-creation of electrons in GaSe and their collection at the Au-electrode. Right: Responsivity *R* versus the average power *P* incident on the sensor (*P* = *Ef*_rep_). **bi** Sensor response to a single pulse within a train of pulses with different *f*_rep_ at *E* = 0.31 nJ. **bii** Left: *Q* versus *f*_rep_ at *E* = 0.31 nJ. Right: *R* versus *P* at *E* = 0.31 nJ

**Fig. 4:**
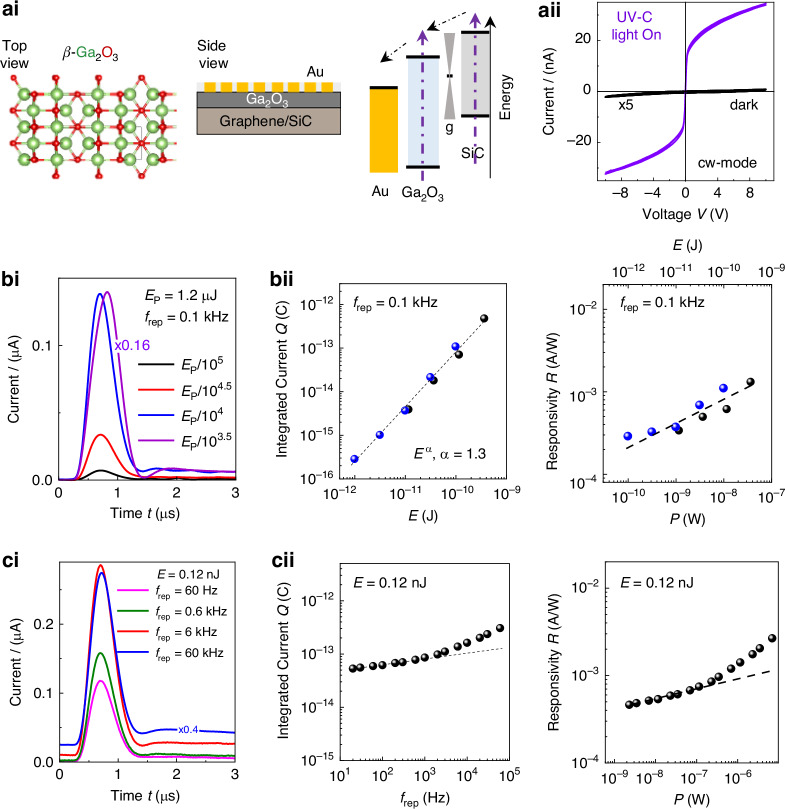
Detection of fs UV-C pulses by Ga_2_O_3_/graphene/SiC. **ai** Schematic of the Ga_2_O_3_ crystal lattice (in-plane view) and of the Au/Ga_2_O_3_/graphene/SiC sensor (side view) and its excitation by laser pulses. **aii** Current-voltage characteristics of the sensor in the dark (black) and under cw UV-C illumination (violet) from a Xe-lamp (*P* = 3.3 μW, *λ* = 260 nm). **bi** Sensor response to a single UV-C (256 nm) pulse within a train of pulses at different energies *E* and fixed repetition rate (*f*_rep_ = 0.1 kHz) at *V* = 2 V in air. **bii** Left: Integrated current *Q* versus *E*. The dashed line describes a super-linear dependence of *Q* on *E*. Different colors correspond to repeated experiments. Right: Responsivity *R* versus *P*. The dashed line is a guide to the eye. **ci** Sensor response to a single UV-C (256 nm) pulse within a train of pulses with different *f*_rep_ and *E* = 0.12 nJ in air (*V* = 2 V). **cii** Left: *Q* versus *f*_rep_ at *E* = 0.12 nJ. Right: *R* versus *P*. Dashed lines are guides to the eye

The UV-C sensors are based on GaSe (Figs. 2 and 3) or its oxide Ga_2_O_3_ (Fig. 4) whose layer thickness *l* is well controlled and tuneable. As discussed in the following, both materials possess electronic properties that are well suited for strong light-matter interaction in the UV-C range.

The GaSe crystals were grown by molecular beam epitaxy (MBE) on sapphire (Fig. 2a) or on graphene/SiC substrates (see Methods and Supplementary Note 2). The single GaSe layer features a hexagonal lattice and an atomic configuration corresponding to a dominant D_3*d*_ centrosymmetric polymorph (Fig. 2a): each van der Waals (vdW) layer consists of four layers of atoms in the sequence Se-Ga-Ga-Se along the *c*-axis, perpendicular to the layer plane; the Ga-Se bonds in the upper and lower part of the centrosymmetric vdW layer are rotated by 60^∘^. The single or multiple vdW layers represent a photoactive medium with a large optical absorption coefficient in the UV-C (> 10^6^ cm^−1^ for *λ* < 265 nm) and bandgap energy *E*_g_ that increases from 2.1 eV (direct bandgap) to 3.5 eV (indirect bandgap) with decreasing layer thickness *l* down to the single vdW layer (*l* = 0.8 nm)^27,28^. The VIS band edge absorption in GaSe arises from band states in the conduction and valence bands with opposite parity (e.g. s- and p-like band states), coupling only weakly with light polarized in the layer plane; in contrast, in the UV-C range, the absorption arises from states with the same parity and is stronger under the in-plane polarized light used in this experiment^27^. In thin-films of GaSe, only the upper layers absorb UV-C light due to the small absorption length (<10 nm) in this spectral range. Post-growth oxidation of GaSe in an oxygen-rich environment at temperatures *T*_a_ of up to 800°C (Figure S2 in Supplementary Note 2) was used to promote the chemical transformation of GaSe into *β*-Ga_2_O_3_, which has a bandgap energy *E*_g_ = 4.5 eV (*λ* = 275 nm) at 300 K. This approach improves the spectral selectivity of the sensor in the UV-C, although the absorption coefficient of *β*-Ga_2_O_3_ (10^4^ cm^−1^) is smaller than for GaSe (10^6^ cm^−1^) at *λ* = 256 nm^34^.

### Detection of fs UV-C laser pulses by GaSe

We now focus on the response of representative sensors to fs UV-C pulses. These studies differ from previous work on exfoliated flakes of GaSe^35^ and/or photodetectors that use epitaxial 2DSEM excited by long (ms) light pulses^27,36^ or longer *λ* in the VIS/UV-A/UV-B ranges^36^. Also, our materials are scalable and therefore compatible with the fabrication of large area detectors. The sensors comprise interdigitated Au electrodes (finger spacing of 50 μm) on the photoactive GaSe and have sensing area *A* of 1 mm^2^ (Fig. 2b). A shadow mask was used to pattern the electrodes, avoiding the use of polymers and hence the degradation of the layers during lithography, etching, and contact deposition. Our metal-semiconductor-metal photodetectors contain two Schottky contacts. They demonstrate electrical insulation in the dark and photoresponse under light, as tested in the cw and pulse mode using different sources of UV-C light (LED and Xe-lamp), see Methods and Figs. S3–S4 in Supplementary Notes 3–4.

Figure 2c shows a circuit model of the sensor and its response to a train of fs UV-C pulses. The photocurrent signal from the sensor is amplified and converted to a voltage *V* by a trans-impedance amplifier with a gain of 10^7^ VA^−1^ and a bandwidth of 0.5 MHz. The amplified signal is then sampled by a high-speed oscilloscope. Since the photocurrent is proportional to the incident power, the train of fs laser pulses results in a train of short electrical pulses. The temporal dependence of each electrical pulse arises from the convolution of the optical pulse with the detector’s response, which is determined by the RC-time constant (μs) of the measurement circuit.

The electrical pulses were measured for different pulse energies *E* (Fig. 3ai) and repetition frequencies *f*_rep_ (Fig. 3bi). To avoid contamination of the sensor in air, all experiments were conducted in vacuum (pressure of 10^−6^ mbar). The average photocurrent $\bar{I}$ under a train of laser pulses is calculated as $\bar{I}$ = *Qf*_rep_, where *Q* is the integrated photocurrent over one period *T* = 1/*f*_rep_, e.g. *Q* = ∫*I*(*t*) *dt*. As shown in Fig. 3aii, the value of *Q* increases linearly with *E*. Correspondingly, the responsivity, *R* = *Q*/*E*, remains constant at different *E*. Also, as shown in Fig. 3bii, at fixed *E*, the values of *Q* and *R* remain approximately constant with increasing *f*_rep_ (or *P* = *Ef*_rep_), but decrease at high *f*_rep_ (>10 kHz).

The detection of the UV-C laser pulses by the GaSe sensor relies on the generation of electron-hole pairs, their separation by the applied electric field, and their extraction at the Au electrodes (inset of Fig. 3aii). The fast temporal response of the sensor arises from the short lifetime (<1 ns) of the photocreated carriers in GaSe^37,38^ and it is limited at high *f*_rep_ by the RC-time of the measuring circuit. The GaSe sensors can operate in vacuum and air. However, their performance can be affected by thermal heating at high energy pulses (*E* > 1 μJ) and/or at high repetition rates. This is not surprising, as a train of fs laser pulses can carry a large power: a single (243 fs) pulse with energy *E* = 1 μJ corresponds to a peak power *P* = 4 × 10^6^ W. This can induce a release of heat that cannot be dissipated efficiently by the sensor. Thermal heating depends on the pulse energy and repetition rate (Supplementary Note 3) and it can be inferred from the reversible rapid (on ms timescales) reduction of signal after the detection of the first laser pulse (Fig. S5 in Supplementary Note 5). Heating effects could be avoided by operating the sensor at low *E* and by thermal management. Also, the exposure of the GaSe surface to air and its excitation by high-energy laser pulses can contribute to a gradual, irreversible loss of photocurrent signal over time due to oxidation of the GaSe surface. Under light, the photoexcited electrons are transferred to oxygen molecules and generate superoxide anions that react with GaSe, leading to the conversion of Ga-Se bonds into Ga-O bonds, followed by the formation of various by-products, such as amorphous-Se, Ga_2_Se_3_ and Ga_2_O_3_^34^. Oxidation is particularly sensitive to UV light as a result of the high photon energies and the large absorption of GaSe in the UV. Although oxidation can limit the operation of GaSe in air, it can also be exploited for the full conversion of GaSe into Ga_2_O_3_. Here, we focus on a sensor based on the controlled oxidation of a 5 nm-thick GaSe layer on graphene/SiC. This is achieved through thermal annealing of GaSe in oxygen at *T*_a_ = 800 °C (Fig. 4 and Supplementary Note 2).

### Detection of fs UV-C laser pulses by Ga_2_O_3_/graphene/SiC

The Ga_2_O_3_/graphene/SiC sensor (Fig. 4ai) displays insulating behavior in the dark and a large on/off current ratio under UV-C light in pulse and cw (Fig. 4aii) modes. It can operate at low applied voltages *V* with a small dark current (<0.1 nA) that is weakly dependent on *V*. The photocurrent increases with increasing *V*, but tends to saturate at large *V*, and increases with decreasing *λ* below 350 nm (Fig. S4 in Supplementary Note 4). Under illumination with fs UV-C laser pulses (Fig. 4bi), the integrated photocurrent *Q* exhibits a super-linear dependence on the pulse energy *E* with a coefficient *α* = 1.3, resulting in an increase of *R* with increasing *E* or *P* (Fig. 4bii). Also, at fixed *E*, the values of *R* and *Q* tend to increase with increasing *f*_rep_ or *P* (Fig. 4ci and 4cii). Thus, compared to GaSe, the behavior of the Ga_2_O_3_/graphene/SiC heterostructure is qualitatively different with a responsivity that increases with *E*, *P*, and *f*_rep_. The dynamics of the pulse train is also different: the amplitude of the pulses detected by the sensor tends to increase rapidly (on ms timescales) after the detection of the first pulse (Figures S5–S6 in Supplementary Note 5).

The increase of responsivity at large *E* or *P* is unexpected. In fact, most sensors are less efficient at high powers because of an increasing carrier recombination rate, causing a sub-linear *P*-dependent photocurrent and loss of performance. A super-linear *P*-dependent photocurrent can arise from different mechanisms. First, we examine nonlinear optical processes, such as two-photon absorption (TPA). Using the small TPA coefficient for Ga_2_O_3_ in the UV^39,40^, we estimate that the laser power required to induce a significant TPA-induced increase in the responsivity is several orders of magnitude larger than that used in our study (<1 GWcm^−2^, Supplementary Note 4). However, we note that photo-thermionic effects at the interface of graphene with a 2DSEM can contribute to super-linear behaviors^41^. The super-linearity reported in previous hybrid graphene-2DSEM structures is generally weak with *P*-law exponents that are smaller (*α* < 1.1) than those measured in our Ga_2_O_3_/graphene/SiC device (*α* = 1.3). Since we observed a weaker super-linear *P*-dependence in structures with a thicker (30 nm) Ga_2_O_3_ on graphene/SiC (Figure S7–S8 in Supplementary Note 6), we propose that the super-linear behavior can partially arise from a light-induced thermal injection of photocreated carriers from the graphene/SiC into the Ga_2_O_3_ channel. In particular, the *P*-dependent occupancy of carrier recombination centers can play a crucial role in the photoresponse. In most semiconductors, sub-linear *P*-dependent photocurrents at large powers arise from radiative, Auger, and level-trap recombination of the photocreated carriers. Super-linear photocurrents are less common, but can be accounted for by recombination centers with a *P*-dependent occupancy and/or capture absorption cross-sections^42–44^: With increasing power, the centers can saturate, thus closing a path for the recombination of the photocarriers and increasing their lifetime. These centers can exist in the Ga_2_O_3_ layer as well as at its interface with the graphene/SiC substrate. Intrinsic and extrinsic defects, such as O- and Ga-vacancies in Ga_2_O_3_, and their power-dependent charged states can affect not only the capture and recombination (radiative and non-radiative) of photoexcited carriers, but they can also increase carrier absorption cross-sections^45^. This behavior is expected to be more likely in a wide bandgap semiconductor, such as Ga_2_O_3_ or SiC, for two main reasons. First, a wide bandgap semiconductor possesses a smaller concentration of free carriers; second, there are fewer carriers occupying discrete states within the bandgap prior to their generation by light^44^.

## Discussion

This work combines for the first time the generation of fs UV-C laser pulses with their fast detection by two-dimensional semiconductors. We have exploited phase-matched second-order processes in nonlinear optical crystals for efficient generation of UV-C laser light. Optimization of crystal thicknesses in the BiBO/BBO system leads to a modular cascaded fourth harmonic generation with a conversion efficiency of 20% from the NIR to the UV-C. The SHG efficiency scales approximately quadratically with the crystal thickness. However, for short pulses, the group velocity mismatch between the fundamental and second harmonic causes temporal walk-off, limiting the usable thickness. Additional constraints arise from back-conversion and two-photon absorption of the second harmonic. The high conversion efficiency marks a significant milestone and provides a foundation for further optimization and scaling of the system into a compact UV-C source.

We have presented two approaches to detection of the fs UV-C laser pulses by 2DSEM. These include the 2DSEM GaSe with strong resonances in the UV-C and its oxide, Ga_2_O_3_, with band edge absorption in the UV-C range. Both materials can be fabricated at scale by state-of-the-art manufacturing technologies and their electronic properties are well-suited for low-power sensors along with a fast temporal response. Also, both sensors exhibit an increase of the photocurrent with increasing pulse energy or power. However, their specific power dependence is qualitatively different. For GaSe on sapphire, the photocurrent exhibits a linear *P*-dependence. In contrast, for Ga_2_O_3_ on graphene/SiC, the *P*-dependence is super-linear with a power coefficient *α* = 1.3. This points to photo-thermionic effects and a *P*-dependent occupancy of defect states in the Ga_2_O_3_/graphene/SiC heterostructure. These 2DSEM sensors can detect not only UV-C fs pulses but also have reliable performance over broad ranges of pulse energies and pulse repetition rates. To date, this capability has not been demonstrated with traditional semiconductors or 2DSEM. In general, while the sensor responsivity is a performance parameter reported in the literature, the temporal response is often omitted. In particular, a sensor with a large responsivity tends to suffer from a slow temporal response, a correlation associated with photo-gain effects due to charge traps^46^.

**Fig. 5:**
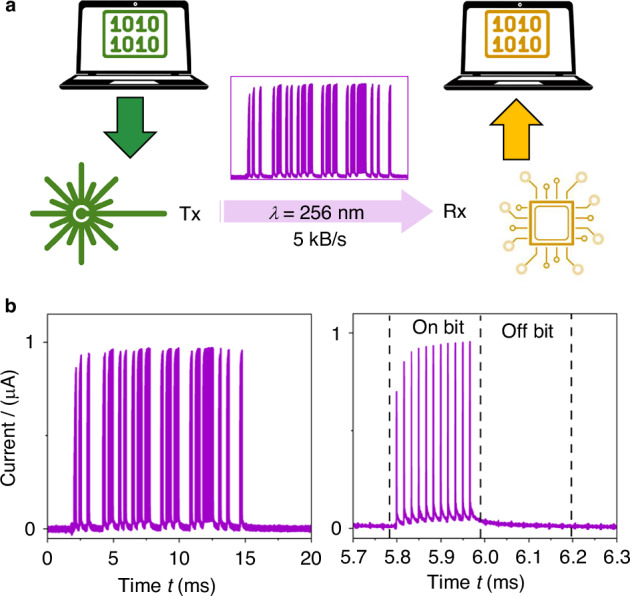
Free-space UV-C communication by integration of UV-C source and sensor. **a** A message is coded by the UV-C laser source-transmitter (green) and decoded by the sensor-receiver (yellow). The arbitrary waveform generator function of a Moku: Go data acquisition card (DAC) is used to generate a binary voltage signal that encodes an ASCII message via the universal asynchronous receiver-transmitter (UART) protocol at a bit rate of 5 kHz. The UV-C (256 nm) femtosecond laser pulses (*f*_rep_ = 60 kHz, *E* = 0.1 nJ) are modulated using the 5 kHz UART signal. The message is transmitted in free space and received by a Ga_2_O_3_/graphene/SiC sensor (at 3 m from the source in line of sight). The signal is recorded by streaming the voltage output to the data logger software on a Moku:Go DAC. **b** Left: Electrical pulses decoded by the sensor. Right: Individual pulses within the train of pulses shown on the left

The generation and detection of UV-C laser pulses demonstrated in this work can dramatically impact many applications. With the rise of compact, robust, and efficient lasers that are capable of delivering fs pulses with several watts of average power in the NIR, the generation of high-order harmonics presents a promising route for generating laser pulses at specific wavelengths in the UV-C range. The availability of fast laser pulse counting detectors is a prerequisite for all applications. The sensing capabilities of GaSe (from the VIS to the UV-C) and Ga_2_O_3_ (UV-C) can stimulate the development of new integrated source-sensor platforms for specific applications, such as free-space communication between autonomous systems and robotics, where machine-to-machine communication is key. This is shown in Fig. 5 where a message is coded by the UV-C laser source-transmitter and decoded by the sensor-receiver (see details in the Methods section). In addition, our metal-semiconductor-metal photodetectors have a simple planar structure and are suitable for monolithic integration with other components on photonic integrated circuits. For example, patterned oxidation of GaSe could be directed towards the fabrication of self-powered GaSe/Ga_2_O_3_ junction devices with spectral tunability and imaging capability. Both the GaSe and Ga_2_O_3_-based devices in this work offer a promising route towards commercialization. The manufacturing of our materials and their processing are scalable and affordable. Different routes to manufacturing could include other cheaper, but less controlled synthetic methods, such as chemical vapour deposition. Finally, with the efficient generation of UV-C laser light by nonlinear optical processes, we envisage a versatile use of the proposed source-sensor system for different technologies, spanning from broad-band imaging to spectroscopy on femtosecond timescales.

## Methods

### Materials

#### Generation of fs UV-C laser pulses

The 1 mm-thick anti-reflection coated BiBO crystal (Crystech) is cut at an angle of 166.6^°^ for type 1 eeo phase-matched SHG to produce light at 512 nm. An anti-reflection coated 0.3 mm-thick BBO crystal (Eksma Optics BBO-644H) was placed 5 mm after the BiBO crystal. The BBO is cut at an angle of 50^°^ for type 1 ooe phase-matched SHG to generate the FH at 256 nm. The crystal thickness is a key parameter influencing the conversion efficiency. We used numerical simulations^47^ (via Lightwave Explorer package at https://github.com/NickKarpowicz/LightwaveExplorer) to determine the optimal crystal thickness.

#### Detection of fs UV-C laser pulses

The GaSe layers were grown by MBE on 2 inch *c*-plane (0001) sapphire or on 1 cm^2^ epitaxial graphene/SiC substrates using a Scienta Omicron PRO 75 MBE system. For the thermal conversion of GaSe into an oxide, we used a Carbolite Gero TF1 12/125/400 tube furnace. The oxidation was conducted at a temperature of up to 800 ^∘^C under an argon/oxygen gas mixture, as required to produce crystalline Ga_2_O_3_. Details of the growth and post-growth oxidation studies of GaSe are in the Supplementary Note 2.

### Techniques

#### Electrical measurements

The photoresponse of the sensors was examined under excitation by fs UV-C laser pulses at fixed wavelength *λ* = 256 nm. Preliminary studies were also conducted using an ams OSRAM UV-C LED (*λ* = 265 nm) and a Xe-lamp (Supplementary Notes 3, 4). The Xe-lamp was used to probe the spectral response of the sensor with wavelengths in the VIS and UV selected by a HORIBA Jobin Yvon MicroHR monochromator. The power of light was controlled using UV-compatible neutral density optical filters and measured using a Thorlabs PM100D power meter. The source-drain voltage was supplied by a Keithley 2400 SourceMeter, which also measured the current through the device for the current-voltage characterisation and responsivity measurements. For the time resolved photocurrent measurements, the current was amplified by a NF SA-604F2 Wideband Current Amplifier, which output to a Yokogawa DL850 Scopecorder. For the detection of the pulses, a number of averages were taken on the oscilloscope to resolve the individual peaks from the background noise.

#### Raman studies

The Raman studies of the GaSe and Ga_2_O_3_ layers (Supplementary Note 2) were carried out using a Horiba Jobin Yvon LABRAM optical confocal microscope system. The experimental setup comprises of a He-Ne (*λ* = 633 nm) laser and a frequency-doubled Nd:YVO_4_ (*λ* = 532 nm) laser, an x-y-z motorized stage and an optical confocal microscope system equipped with a 0.5 m-long monochromator and two gratings (1200 grooves/mm and 150 grooves/mm). The signal was detected by a Si charge-coupled device camera. The laser beam was focused onto the sample down to a spot diameter of ~1 μm using a 100 × objective.

#### UV-C Communication

The arbitrary waveform generator function of a Moku:Go data acquisition card (DAC) was used to generate a binary voltage signal that encoded an ASCII message via the universal asynchronous receiver-transmitter (UART)^48^ protocol at a bit rate of 5 kHz. This voltage signal was used to control the pulse picker of the Carbide laser (operating at *f*_rep_ = 60 kHz), which allowed the binary digits to be modulated onto the pulse train generated by the laser. This resulted in a binary signal being represented by a sequence of 12 UV-C pulses. The UV-C beam was transmitted across 3 m of free space where it was detected by a Ga_2_O_3_/graphene/SiC sensor. A NF SA-604F2 wideband current amplifier was used to generate an amplified voltage signal, which was recorded by the data logger function of the Moku:Go DAC. A voltage threshold was used to determine whether the sensor was in the “ON” state (1) or the “OFF” (0) state. The string of 1 and 0 s was separated into the individual characters (8 bits) using the pre-determined data transmission rate (5 kHz) and finally converted back to the associated ASCII characters by a Python script.

## Supplementary information

Supplemental Material

## Data Availability

The data that support the findings of this study are available from the corresponding author upon reasonable request.
