# Supplementary material for: Fast ultraviolet-C photonics: generating and sensing laser pulses on femtosecond timescales
Source: Light Sci Appl. 2025 Nov 19;14:384. doi: 10.1038/s41377-025-02042-2 (PMC12627855; doi:10.1038/s41377-025-02042-2)
Supplement: Supplementary file 1 — Supplemental Material [file 41377_2025_2042_MOESM1_ESM.docx]

# Supplementary Information

**Fast Ultraviolet-C Photonics:**

**Generating and Sensing Laser Pulses on Femtosecond Timescales**

*Benjamin T. Dewes^1^, Tim Klee^2^, Nathan D. Cottam^1^, Joseph J. Broughton^2^, Mustaqeem Shiffa^1^*, *Tin S. Cheng^1^, Sergei V. Novikov^1^, Oleg Makarovsky^1^,* *John W. G. Tisch^2*^, and Amalia Patanè^1*^*

^1^ School of Physics and Astronomy, University of Nottingham, Nottingham, NG7 2RD, UK

^2^ Blackett Laboratory, Imperial College London, London SW7 2AZ, UK

*Author to whom any correspondence should be addressed

[amalia.patane@nottingham.ac.uk](mailto:amalia.patane@nottingham.ac.uk); [john.tisch@imperial.ac.uk](mailto:john.tisch@imperial.ac.uk)

**Table of contents**

**1. Generation of fs UV-C laser pulses**

**2. Materials for UV-C sensing**

**3. Sensors based on gallium selenide on sapphire**

**4. Sensors based on thin-layer gallium oxide on graphene/SiC**

**5. Temporal response of UV-C sensors**

**6. Sensors based on thick-layer gallium oxide on graphene/SiC**

**List of Figures**

**Figure S1:** Properties of fs UV-C laser pulses

**Figure S2:** Raman spectra of 30 nm-thick GaSe on graphene/SiC before and after annealing

**Figure S3:** Photoresponse of a 55 nm-thick GaSe/sapphire sensor to fs UV-C laser pulses

**Figure S4:** Photoresponse of 5 nm-thick Ga_2_O_3_ on graphene/SiC

**Figure S5:** Temporal response of GaSe and Ga_2_O_3_ sensors

**Figure S6:** Temporal response of 5 nm-thick Ga_2_O_3_ sensors

**Figure S7:** Photoresponse of 30 nm-thick Ga_2_O_3_ on graphene/SiC: cw-mode

**Figure S8:** Photoresponse of 30 nm-thick Ga_2_O_3_ on graphene/SiC: pulsed-mode

**1. Generation of fs UV-C laser pulses**

The generation of 256 nm pulses was achieved via the fourth harmonic (FH) of a Yb laser (CB5, Carbide). The fundamental has a central wavelength of 1024 nm with a

full width at half maximum (FWHM) of 4.3 nm, measured using a commercial spectrometer (USB2000+, Ocean Optics). The temporal FWHM of the fundamental is 243 fs, measured using autocorrelation (assuming a Gaussian pulse envelope). The spatial mode of the fundamental is Gaussian with a near-diffraction limited beam quality factor of 1.04. The laser repetition rate, *f*_rep_, was set to 60 kHz. An integrated Pockels cell was used as a pulse picker, allowing for the repetition rate of the emitted beam to vary from 0 up to 60 kHz (by any integer divisor of the base repetition rate).The experimental setup of the FH generation system is described in the main manuscript. Here, we provide further details.

**1.1 Experimental setup**

Using a beamsplitter (BFS10-B1, Thorlabs), 7.9% of the fundamental was reflectively split off to be used in the cross-correlator. This was then used to measure the FH pulse duration. An achromatic half-waveplate (467-4215, Eksma Optics) and thin film polariser (420-1268E, Eksma Optics) were employed to ensure the fundamental laser pulses were s-polarised and to allow for control of the pulse energy. A 1 m focal length lens (KPX124, Newport) was used to focus the initially collimated 2.35 mm 1/*e*^2^ diameter fundamental to a spot size of 498 μm, as measured with a beam profiler (BC106N-VIS/M, Thorlabs). A 1 mm thick anti-reflection coated BiBO crystal (Crystech) was placed at the focus, which is cut at 𝜃 = 166.6° for type 1 eeo phase-matched second harmonic generation (SHG) to produce 512 nm light (the second harmonic, SH). A 6-axis mount (K6XS, Thorlabs) allows for the positioning of the crystal to ensure phase matching. An anti-reflection coated 0.3 mm thick BBO crystal (BBO-644H, Eksma Optics) in an identical 6-axis mount was then placed 5 mm after the BiBO crystal. The BBO is cut at 𝜃 = 50° for type 1 ooe phase matched second harmonic generation to generate the FH at 256 nm from the incident SH. Note that this configuration of crystals for FH generation is a non-standard configuration compared to those reported in the literature. Usually, the co-propagating fundamental and SH are allowed to diverge after the initial nonlinear crystal, collimated via a lens, and then a dichroic mirror is used to separate the two wavelengths. In this work the fundamental is still present in the second crystal that produced the FH. However, as no nonlinear processes involving the fundamental are phase matched, the FH process is not significantly affected.

Two harmonic separators (042-2535PHT, Eksma Optics) were used at a 45° angle of

incidence to isolate the FH. Their dielectric thin layer coating allows for a 99.5% reflectivity for the s-polarised FH, whilst having a transmission of 98.5 % for both the SH (p-polarised) and the fundamental (s-polarised). A different harmonic separator (042-5135, Eksma Optics) was used to separate the SH from the fundamental.

The SH average power 𝑃_SH_ with the BBO crystal removed was measured using a power meter (3A-P, Ophir) as the fundamental average power 𝑃_F_ was varied from 0 to 1.267 W with *f*_rep_ = 60 kHz. The SH pulse energy increases with the fundamental pulse energy, **Figure S1a**. Similarly, with the BBO in place, the FH average power 𝑃_FH_ was measured as 𝑃_F_ was varied over the same range. The FH pulse energy increases with the fundamental pulse energy, **Figure S1b**. A saturation behaviour is observed only at the largest pulse energies. Note that 𝑃_F_ was measured directly after the lens, whilst 𝑃_SH_ and 𝑃_FH_ were measured after their respective harmonic separators. For 𝑃_F_ = 705.8 mW (corresponding to a pulse energy of 11.8 μJ), the fundamental to FH conversion efficiency peaked at 20.27%. The SH spectrum was measured using a spectrometer (USB2000+, Ocean Optics). The SH central wavelength is 512 nm with a FWHM of 2.2 nm. Using a higher resolution spectrometer (VS7550, Resonance), the FH spectrum was also measured, giving a FH central wavelength of 256 nm with a FWHM of 0.52 nm. The FH spatial profile of the diverging beam (divergence half angle of 0.45 mrad) was measured via a CCD camera (a2A4504-18umBAS, Basler), see inset of **Figure S1b**. The spatial profile is Gaussian with a 1/*e*^2^ diameter of 895 μm.

**Figure S1** a)Dependence of the second harmonic (SH) pulse energy on the fundamental pulse energy. b) Dependence of the fourth harmonic (FH) pulse energy on the fundamental pulse energy. Inset: Spatial profile of the FH at the position of the UV-C sensor tested in this work.

**1.2 Materials for UV-C generation**

Different combinations of materials can be used for FH in the UV-C.

The thickness of the BiBO and BBO crystals is a key parameter influencing the conversion efficiency. We used numerical simulations (via Lightwave Explorer package at https://github.com/NickKarpowicz/LightwaveExplorer) to determine the optimal crystal thickness considering different competing effects. The SHG efficiency scales approximately quadratically with the crystal thickness. However, for short pulses (as in our case), the group velocity mismatch between the fundamental and second harmonic causes temporal walk-off, limiting the usable thickness. Additional constraints arise from back-conversion and two-photon absorption of the second harmonic.

Using a second BiBO crystal for the final SHG step to reach 256 nm would not be appropriate as the BiBO’s effective nonlinearity drops to zero near 515 nm (see [1], Fig. 8a). BBO can phase-match SHG for a 1024 nm fundamental and would likely give comparable efficiency to BiBO in the first stage under ~10 μJ pulse energies. For example, [2] reports ~50% NIR-to-green conversion with BBO, while we observe >60% using BiBO. However, we chose BiBO for the first crystal to enable compatibility with compact, low-pulse-energy lasers (e.g. ~100 nJ from fiber lasers). In that regime, tight focusing is needed to reach the required intensities for SHG (see Main Paper Ref. [30]). BiBO, with ~1.5× higher nonlinearity than BBO at 1024 nm, is better suited to low-energy conditions, especially where spatial walk-off is significant. BBO's NIR-to-green efficiency plateaus at 51% [3], below what BiBO can achieve. We note further that optical parametric amplifiers (OPAs) can also produce tunable fs UV-C pulses, but they typically required relatively complex and larger pump sources (e.g. Ti:sapphire chirped pulse amplification systems) which would not be compatible with our goal of developing a compact and portable source.

**2. Materials for UV-C sensing**

We have used molecular beam epitaxy (MBE) to grow GaSe layers on Al_2_O_3_ (sapphire) and on commercial (Graphensic) graphene/SiC substrates. Details of the MBE setup, growth parameters and properties of GaSe layers can be found in references [4-5]. The grown GaSe layers were used for development of UV-C sensors and for post-growth oxidation studies of GaSe. For the sensors, we focus on GaSe on Al_2_O_3_ and GaSe on graphene/SiC with GaSe layer thickness of about 50 and 5 nm, respectively [4-5]. The choice of the layer thickness for the two sensors is dictated by the quality of the grown materials, as probed by Raman spectroscopy, scanning probe microscopy and/or angle-resolved photoelectron spectroscopy. Thinner GaSe layers exhibit better quality on graphene/SiC, whereas the opposite applies to GaSe on sapphire.

For the post-growth oxidation studies of GaSe, thermal annealing was performed in a tube furnace under a controlled O (0.5 sLmin^-1^) and Ar (2.0 sLmin^-1^) atmosphere. Using annealing temperatures *T*_a_ = 400, 600, 800 °C and annealing time *t*_a_ = 30 minutes, we have aimed at the conversion of GaSe into Ga_2_O_3_. The optical characterization of thin layers of Ga_2_O_3_ on graphene/SiC presents a number of challenges compared to Ga_2_O_3_ on sapphire due to the non-transparent SiC substrate and the influence of the oxidation on graphene. We have used Raman spectroscopy to probe the modification of the GaSe and graphene layers following the thermal oxidation. For this study, we examined GaSe samples on graphene/SiC with different layer thickness *l* = 5, 10 and 30 nm and a control graphene/SiC sample.

**Figure S2a** shows the Raman spectra for a GaSe layer (*l* = 30 nm) before and after annealing at temperatures ranging from *T*_a_ = 400 °C to 800 °C and annealing time *t*_a_ = 30 minutes. In the pristine GaSe layers, the A^1^_1g_ and A^2^_1g_ peaks of GaSe are observed at 132 cm^-1^ and 308 cm^-1^, respectively, as expected for a dominant centrosymmetric (D_3d_) polymorph of GaSe. The annealing at *T*_a_ = 400 °C produces an additional peak at 155 cm^-1^, suggesting partial conversion of the GaSe layers into Ga_2_Se_3_. Increasing the annealing temperature to *T*_a_ = 600 °C and 800 °C leads to the disappearance of the GaSe and Ga_2_Se_3_ Raman peaks. The Raman modes due to *β-*Ga_2_O_3_ (at 201 cm^-1^ for the A^3^_g_ mode and at 347 cm^-1^the A^5^_g_ mode) are observed for thermal oxidation of GaSe on sapphire [6] but are masked here by the dominant contribution of the Raman signal from the SiC substrate.

**Figure S2** a)Raman spectra of 30 nm-thick GaSe on graphene/SiC before and after annealing at various temperatures *T*_a_ from 400°C to 800°C and annealing time *t*_a_ = 30 minutes. The Raman spectra were measured under illumination with laser light of power *P* = 0.07 mW and wavelength * *= 633 nm (~1 μm spot size). Since no Raman signal from GaSe or its by-products could be measured following the thermal annealing at 600 and 800 °C, we also measured the Raman spectra under illumination with laser light of power *P* = 23 mW and shorter * *= 532 nm (blue curve). b) Raman spectra for the same sample shown in part (a), but for the spectral region of the G and 2D peaks of graphene. All spectra were measured under illumination with laser light of power *P* = 2.3 mW and * *= 532 nm. The SiC background has been subtracted to reveal the Raman peaks of graphene.

We have examined the Raman spectra of the GaSe/graphene/SiC samples before and after each thermal annealing, focusing on the frequency range of the graphene Raman peaks. Increasing *T*_a_ causes a weakening or quenching of the G and 2D peaks of graphene (**Figure S2b**). A broadening and downshift of these peaks is observed at *T*_a_ = 600 °C, suggesting a strained graphene layer. In general, compressive strain results in an upshift of all Raman peaks of graphene due to the shortening of the C-C bond lengths, while tensile causes a downshift due to the elongation of the bonds [7]. The Raman spectra do not reveal any D peak, whose intensity should increase relative to that of the G or 2D peaks with increasing disorder. We observed a similar trend in samples with thinner GaSe (*l* = 5 and 10 nm) and in a control graphene/SiC sample. We propose that the graphene layer is modified by oxygen-containing functional groups, such as epoxide (C–O–C) and carboxyl (C = O) groups [8-9].

**3. Sensors based on gallium selenide on sapphire**

The detection of photons in the GaSe/sapphire sensors relies on the separation of photo-carriers by the applied electric field across a GaSe channel whose effective thickness is determined by the absorption length *l*_abs_ of GaSe. From the measured absorption spectrum of GaSe in the UV-C range, we estimate *l*_abs_ =  nm at *λ* =  nm [4]. The photoresponse of the sensors was investigated for different applied voltages (*V* = 2 V and 10 V) and under excitation by UV-C fs laser pulses at fixed wavelength *λ* =  nm in vacuum (Figure 3 in the main text) and in air (**Figure S3**). Also, the spectral response was investigated using an UV-C LED (ams OSRAM) at *λ* = 265 nm, and a Xe lamp with wavelength tuneable from the VIS to the UV-C, as described in references [4] and [6]. Studies with the Xe lamp reveal absorption peaks in the UV-C range. **Figure S3** shows the response of a GaSe/sapphire sensor to fs UV-C laser pulses. The sensor was measured in air over a range of pulse energies *E* and pulse repetition rates *f*_rep_. It can be seen that at high *E*, the photocurrent signal exhibits a sublinear dependence on *E*, causing a lower responsivity (**Figure S3a**). Also, at fixed energy, the photocurrent signal tends to decrease with increasing *f*_rep_ (**Figure S3b**). The lower responsivity at high *E, P* or *f*_rep_ is attributed to the release of heat that is not efficiently dissipated by the sensor (**Figure S3c**).

**Figure S3** ai) Response of GaSe (55 nm-thick) in air to a single pulse within a train of pulses at different energies *E* and repetition rate *f*_rep_ (*V* = 10 V) in air. aii) Left: Responsivity *R* versus the average power *P* incident on the sensor. The dashed line is a guide to the eye. Right: Integrated current *Q* versus *E*. The dashed line shows a linear dependence of Q versus *E*. bi) Sensor response to a single pulse within a train of pulses with different *f*_rep_ at a fixed energy *E* = 3.6 nJ. bii) Left: *R* versus *P*. Right: *Q* versus *f*_rep_. c) Schematic of the sensor and its excitation by laser pulses, followed by generation of carriers and their relaxation by radiative and non-radiative processes.

**4. Sensors based on thin-layer gallium oxide on graphene/SiC**

Epitaxial graphene provides an ideal platform for epitaxy of thin layers of GaSe and novel sensor concepts for UV-C. These sensors exploit a charge dipole and a built-in potential at the GaSe/graphene interface, which enables separation of photocreated carriers and transfer of electrons from GaSe into the graphene channel. This mechanism results in photogain and high-responsivity of the graphene channel in the UV-C [5]. However, the photoresponse is limited by the dark conductivity of the graphene/SiC.To reduce the dark conductivity of graphene, we examined sensors produced by post-growth oxidation of GaSe/graphene/SiC. The conductivity properties of the GaSe/graphene/SiC before and after the thermal oxidation were studied at room temperature. Following the oxidation of the GaSe/graphene/SiC sample at *T*_a_ = 800 °C, the dark conductivity decreases by several orders ofmagnitude, from  ~ 10^-4^ Ω^-1^ to 10^-14^ Ω^-1^. This behaviour is also observed in a control graphene/SiC sample and suggests the transformation of the graphene by oxygen-containing functional groups, such as epoxide (C–O–C) and carboxyl (C = O) groups [8-9]. These groups determine the band structure properties of graphene and its conductivity [10]. Additional effects can be envisaged for oxidation at the graphene/GaSe interface, whose vdW gap is likely to be replaced by oxygen-containing groups and lattice deformation at the Ga_2_O_3_/graphene interface.

The photoresponse of a thin-layer (5 nm) Ga_2_O_3_ on graphene/SiC was investigated for different applied voltages and under excitation by UV-C light. The sensor exhibits a larger photoresponse in the UV-C than in the VIS, as probed using a Xe lamp (**Figure S4a**). The bias dependence of the photocurrent shown in Figure 4 of the main manuscript is assigned to a depletion layer effect at the interface between the Au-electrode and the Ga_2_O_3_/graphene/SiC layers (**Figure S4b**): the depletion region extends with increasing the applied voltage until it saturates as the channel is completely depleted; this effect is observed for both negative and positive voltages, and increases the photoresponse as the depletion region widens. We measured the response of the sensor under a temporal modulation of the UV-C LED at voltages *V* = 1V (**Figure S4c**). The 3 dB frequency bandwidth (~ 0.1 MHz) is calculated from the frequency response curve. We calculated the specific detectivity as *D** = sqrt(*A***f*)/NEP ~ 10^10^ Jones, where *A* is the active area of the sensor, *f* is the frequency bandwidth (*f* = 3 dB Bandwidth/0.886) and NEP is the noise equivalent power, defined as the minimum detectable optical power at which the signal-to-noise ratio is 1.

**Figure S4.** a) Dependence of the responsivity *R* on photon wavelength *λ*. b) Schematic of the energy band alignment at the interface of Au with Ga_2_O_3_ under different applied biases. c) Dependence of the responsivity *R* on repetition rate *f*_rep_ (with a 50% duty cycle).

To model the responsivity and the contribution of two photon absorption (TPA), we assume that the detection of photons relies on the separation of photo-carriers by the applied electric field across a Ga_2_O_3_ channel of thickness *l* = 5 nm and absorption length *l*_abs_ (*l*_abs_ = 0.5 μm at *λ* = 256 nm) [6]) and area *A*. We express the responsivity as *R* =[*q*/*hv*][*l*/*l*_α_][*τ*_l_/τ_t_], where *q* = *e* is the electron charge, *hv* = 4.84 eV is the photon energy at *λ* = 256 nm, and *τ*_l_/*τ*_t_ is the ratio between the lifetime of the minority carriers (*τ*_l_) and the transit time (*τ*_t_) of the majority carriers. The measured value of *R* ≈10^-3^-10^-2^ AW^-1^ corresponds to *τ*_l_/*τ*_t_ ≈ 2×10^-3^-2×10^-2^. The correction to *R* by non-optical processes, such as TPA, is expected to be small. By assuming a TPA coefficient *β* = 10^-2^ mGW^-1^ [11-12], we estimate that the change in the value of *R* due to TPA is several orders of magnitude smaller than that measured under the peak powers *P* (< GW) used in this work.

**5. Temporal response of UV-C sensors based on gallium selenide and gallium oxide**

**Figure S5** shows thetemporal dependence of the electrical pulses in the GaSe/sapphire (a) and Ga_2_O_3_/graphene/SiC (b) sensors in response to UV-C fs laser pulses, as probed over time since the arrival of the first laser pulse. With increasing *f*_rep_, the GaSe sensor reveals a quick loss of electrical signal. In contrast, an opposite behaviour is observed in the Ga_2_O_3_ sensor. The two dependencies are assigned to: (a) a sub-linear dependence of the photocurrent on power due to thermal heating in GaSe; (b) a super-linear dependence of the photocurrent on power due to photo-thermionic effects and a saturation of non-radiative recombination centres at high powers.

**Figure S5.** Temporal dependence of the electrical pulses in the GaSe (a) and Ga_2_O_3_ (b) sensors in response to UV-C fs laser pulses, as probed over time since the arrival of the first laser pulse. The panels (i-ii-iii) in parts (a) and (b) show the response for a pulse repetition rate *f*_rep_ = 0.1 kHz (i), 1 kHz (ii) and (iii) 10 kHz at a fixed pulse energy *E*. With increasing *f*_rep_, the GaSe sensor reveals a quick loss of electrical signal. In contrast, an opposite behaviour is observed in the Ga_2_O_3_ sensor.

For the Ga_2_O_3_ sensor, we have estimated the temporal increase of the charge (*Q*) for different pulse repetition rates *f*_rep_ and different average powers *P* = *E f*_rep_ (**Figure S6**). The charge is derived by integration of the current versus time: The integral is calculated over a time interval *Δt* that is equal to twice the characteristic time *τ* associated with the exponential increase of the current (**Figure S6a**). As shown in **Figure S6b**, the charge increases with increasing *P*, but this increase tends to saturate at high *P*, thus suggesting a saturation of trap states filled by the photogenerated carriers. At low *P*, *Q* increases with *P* (*Q* ∝ *P*^0.69^).

**Figure S6.** (a)Temporal dependence of the electrical pulses in the Ga_2_O_3_ sensor in response to UV-C fs laser pulses, as probed over time since the arrival of the first laser pulse (*f*_rep_ = 10 kHz and *E* = 0.1 nJ). The dashed line is an exponential fit to the data, corresponding to a characteristic rise time *τ* = 1.6 ms. (b) The temporal increase of the charge (*Q*) for different average powers *P* = *E f*_rep_ (*E* = 0.1 nJ) for the Ga_2_O_3_ sensor. The dashed line is a guide to the eye.

**6. Sensors based on thick-layer gallium oxide on graphene/SiC**

Here we examine the photoresponse of a thick (30 nm) oxide layer and differences in the device characteristics compared to the thin (5 nm) oxide sensor described in section 4. Firstly, the *I-V* under cw UV-C light shows an exponential increase of the current on the applied voltage *V* (**Figure S7a**). This is different to the *I-V* observed in the thin oxide sensor (Figure 4a in the main manuscript) where a saturation of the *I-V* was observed at large voltages. This behaviour was observed for different light powers and points towards a contribution to the photocurrent signal from both Ga_2_O_3_ and SiC. For thin Ga_2_O_3_, the saturation of the current at high voltage suggests a depletion layer effect at the interface of the Au electrode with the Ga_2_O_3_/graphene/SiC interface. We note that photogenerated carriers can be created in both the Ga_2_O_3_ and SiC layers; these are then collected at the Au-electrodes via the Ga_2_O_3_ layer (**Figure S7b**). The contribution of the Ga_2_O_3_ and SiC layers is evidenced further by the wavelength dependence of the responsivity under cw illumination (**Figure S7c**). The responsivity increases going from the control oxidized graphene/SiC to the Ga_2_O_3_/graphene/SiC samples, with a stronger contribution in the thicker Ga_2_O_3_ layer observed at shorter wavelengths.

**Figure S7** a) Current-voltage characteristics of the Au/Ga_2_O_3_ (30 nm)/graphene/SiC sensor under vacuum in the dark (black) and under cw UV-C light (violet) from a Xe-lamp (*P* = 3.3 μW, *λ* = 260 nm). Schematic of the bi) band diagram and bii) side view of the sensor under UV-C excitation. c) Responsivity *R* versus excitation wavelength (*P* = 0.5 μW) for sensors based on 5 nm Ga_2_O_3_ (red curve), 30 nm Ga_2_O_3_ (blue curve) and the control (grey curve) sensor. Red and green blocks mark ranges where the photon energy is greater than the band gap of Ga_2_O_3_ and SiC, respectively.

The photoresponse under UV-C laser pulses was measured for different pulse energies *E* (**Figure S8a-b**) and repetition frequencies *f*_rep_ (**Figure S8c**). Experiments were conducted in vacuum and air, revealing differences only at the lowest *E*. As shown in **Figure S8a-b**, the value of the integrated current *Q* increases approximatively linearly with *E*. Correspondingly, the responsivity *R* = *Q*/*E* remains approximatively constant at different *E*. Also, as shown in **Figure S8c**, at fixed *E*, the values of *Q* and *R* remain approximately constant with increasing *f*_rep_ but tend to increase at high *f*_rep_.

**Figure S8** ai) Sensor response to a single pulse within a train of pulses at different energies *E* using an unattenuated pulse energy on the sensor *E*_p_ = 0.31 μJ and fixed repetition rate (*f*_rep_ = 0.1 kHz) and *V* = 2 V. aii) Left: Integrated current *Q* versus *E*. The dashed line is a linear dependence of *Q* on *E*. Right: Responsivity *R* versus the average power *P*. The black and red data points in parts ii) correspond to measurements with the sensor in vacuum and air, respectively. bi) Sensor response to a single pulse similar to a) but with *V* = 10 V across the sensor. aii) Left: *Q* versus *E*. The dashed line shows a linear dependence of *Q* on *E*. Right: *R* versus *P*. The black and red data points in parts ii) correspond to measurements with the sensor in vacuum and air, respectively. ci) Sensor response to a single pulse within a train of pulses with different *f*_rep_ and fixed *E* = 0.1 nJ (*V* = 10 V) in vacuum. cii) Left: *Q* versus *f*_rep_. Right: *R* versus *P*. Dashed lines are guides to the eye.

**References**

[1] Petro V. et al. Femtosecond nonlinear frequency conversion based on BiB_3_O_6_, *Laser & Photonics Reviews* **4**, 53-98 (2010).

[2] Kim G. H. et al. Efficient generation of the second and third harmonics of high-power femtosecond Yb:KGW-laser radiation in nonlinear-optical BBO crystals, *Journal of Optical Technology* **83**, 463-467 (2016).

[3] Kim D. J. et al. Optimization of second harmonic generation in a Yb:KGW femtosecond laser, *Japanese Journal of Applied Physics* **57**, 122702 (2018).

[4] Shiffa M. et al. Wafer‐scale two‐dimensional semiconductors for deep UV sensing, *Small* 2305865 (2024).

[5] Bradford J. et al. Epitaxy of GaSe coupled to graphene: From in situ band engineering to photon sensing, *Small* 2404809 (2024).

[6] Cottam N.D. et al. Thin Ga_2_O_3_ layers by thermal oxidation of van der Waals GaSe nanostructures for ultraviolet photon sensing, *ACS Applied Nano Materials* **7** (15) 17553-17560 (2024).

[7] Zabel J. et al. Raman spectroscopy of graphene and bilayer under biaxial strain: Bubbles and balloons, *Nano Lett*ers **12**, 617−621 (2012).

[8] Szabó T. et al. Evolution of surface functional groups in a series of progressively oxidized graphite oxides. *Chem. Mater.* **18**, 2740–2749 (2006).

[9] Zhu Y. et al. Graphene and graphene oxide: synthesis, properties, and applications. *Adv. Mater.* **22**, 3906–3924 (2010).

[10] Roy R. et al. Role of oxygen functionality on the band structure evolution and conductance of reduced graphene oxide. *Chem. Phys. Lett.* **677**, 80-86 (2017).

[11] Tian X. et al. Dispersion of two-photon absorption and nonlinear refraction in β-Ga_2_O_3_ from 350 to 515 nm, *Appl. Phys. Lett*. **124**, 152112 (2024)

[12] Chen H. et al. Characterizations of the nonlinear optical properties for (010) and (-201) beta-phase gallium oxide, *Optics Express* **26** (4), 3938 (2018).
